# Quality of care of patients with type 2 diabetes mellitus at a public sector district hospital

**DOI:** 10.4102/safp.v65i1.5713

**Published:** 2023-06-06

**Authors:** Kelly J. Fredericks, Mergan Naidoo

**Affiliations:** 1 Department of Family Medicine, School of Nursing and Public Health, University of KwaZulu-Natal, Durban, South Africa

**Keywords:** type 2 diabetes mellitus, quality of care, public sector, district hospital, South Africa

## Abstract

**Background:**

Globally, diabetes mellitus (DM) remains one of the leading causes of mortality, with approximately 2 million deaths in 2019, the condition also contributes significantly to adverse health conditions and costs. The study aimed to describe the quality of care (QOC) rendered to patients with type 2 DM (T2DM) seeking care at Wentworth Hospital (WWH), a district hospital in KwaZulu-Natal province, South Africa.

**Methods:**

A descriptive cross-sectional design was used, and all patients living with T2DM on treatment who had accessed care for at least 1 year were included. Data were collected through structured exit interviews, and their clinical data were extracted from their medical records. Their knowledge, attitudes and practices were assessed using a 5-point Likert scale.

**Results:**

The mean age (standard deviation [s.d.]) was 59 (13.0) years and most (65.3%) were female, of African (30.0%) and Indian (38.6%) descent, with two-thirds (69.4%) obtaining a secondary school education. Their mean glycated haemoglobin (HbA1c) (s.d.) was 8.6 (2.4%). Over 82% had one or more comorbidity, while 30% had at least one DM-related complication. Generally, participants were pleased with the care received, but their knowledge and practices related to their T2DM was suboptimal.

**Conclusion:**

This study indicates that the QOC was suboptimal due to poor efficacy indicators, poor knowledge and lack of adequate lifestyle measures, despite the frequency of medical practitioner reviews.

**Contributions:**

This study identified gaps in QOC and will aid South African public sector policy-makers in devising quality improvement initiatives.

## Background

The International Diabetes Federation (IDF) estimated that the global prevalence of diabetes mellitus (DM) has increased by 62% during the past 10 years, from 285 million in 2009 to 463 million.^1^ The most significant relative increase is predicted for Africa, where in 2017, 15.5 million adults had DM, with 69.2% of people being unaware of their DM status.^2^ Most people living with DM in Africa access clinical care at the primary care level in resource-limited settings.^3^ Primary care systems face multiple challenges in delivering DM services, including lack of evidence-based guidelines specific to the population, lack of available medications, differences in urban and rural populations, and inequity between public and private sector healthcare.^4,5^ The public sector in South Africa uses the National Department of Health’s Standard Treatment Guidelines, which is partially based on Society for Endocrinology, Metabolism and Diabetes of South Africa (SEMDSA) treatment guidelines.^6,7^ The large unmet needs of people living with DM necessitate further exploration of patients’ perceptions regarding their care at the primary care level.

South Africa is ranked as an upper-middle-income country and the second-largest economy in Africa. Despite this, it is plagued by high economic and health inequalities due to years of racial and gender discriminatory policies and high levels of unemployment due to low economic growth, which has led to suboptimal public sector health system funding and poor health outcomes often worse than those in poorer countries.^4^ Diabetes mellitus treatment and prevention efforts are further impeded in South Africa by the country’s health system prioritising infectious diseases (e.g. human immunodeficiency virus and acquired immune deficiency syndrome [HIV and AIDS], tuberculosis [TB]) and maternal and child health services. Although TB was still the overall leading cause of natural deaths, in the same time period from 2015 to 2017, DM was the second cause of death, having moved from third rank in 2014.^5^ This further amplifies the countries’ current epidemiologic shift with the increasing prevalence of non-communicable disease (NCDs),^5,8^ which is thought to be largely fuelled by lifestyle changes brought about by a surge in rural-urban migration.^9^

There is overwhelming evidence that healthcare quality in South Africa has been compromised by various challenges that negatively impact healthcare quality.^10^ Improvement in quality care means fewer errors, reduced delays in care delivery, improvement in efficiency, increased market share and at a lower cost.^11^ The decline in the quality of healthcare has caused the public to lose trust in the public healthcare system in South Africa.^10,11^ The Institute of Medicine defines quality in healthcare using six dimensions, namely patient safety, timeliness, efficiency, effectiveness, patients experience of care and equity.^12^

The study aimed to describe the quality of care (QOC) rendered to patients with type 2 DM (T2DM) seeking care at a public sector hospital in Durban, KwaZulu-Natal province, South Africa. Secondary objectives were to review the demographic data and compare participants’ knowledge, attitudes and practices against the SEMDSA treatment guidelines. We define patients’ experience of care and measure process and outcome indicators.

## Methods

The study used an observational cross-sectional study design that assessed the QOC of all patients living with T2DM accessing care in an urban district hospital in Durban, KwaZulu-Natal, South Africa. The study was conducted over 4 months (from 01 February 2020 to 31 May 2020) at the hospital’s chronic outpatient clinic (COPD). The hospital is in the eThekwini District, has 250 inpatient beds and has a catchment population of approximately 333 740. The hospital records show that the staff at the hospital consult an average of 10 600 outpatients per month and admit 764 persons. Medical officers service the hospital’s COPD, which provides ambulatory care to approximately 786 patients living with T2DM monthly. A sample size of 361 was selected for this study (with a power of 95% and a margin of error of 5%). A systematic randomised sampling method was used to select participants, with every third patient meeting the inclusion criteria being asked to participate in the study. Measures were implemented to ensure that participants did not participate in the study more than once, and sampling was stopped when the sample size was attained.

The inclusion criteria were all patients living with T2DM who were 18 years or older and had been attending and receiving care at the hospital’s COPD for 12 months or more. Patients were excluded if they did not consent to participate in the study, were cognitively impaired, pregnant, being treated at other institutions who may have been referred to the researched facility in the past 12 months, had missing clinical files or had defaulted scheduled appointments.

Data were collected using two methods:

- Face-to-face interviews with a structured questionnaire that used open- and closed-ended questions to assess knowledge, attitudes and practices. Interviews were conducted by the principal investigator and a research assistant collector proficient in isiZulu, who was trained and worked with the principal investigator throughout data collection.
- A validated data extraction tool was used to extract information from the participant’s medical records regarding their care over the past 12 months using the SEMDSA treatment guidelines but aligned to the South African Standard Treatment Guidelines developed by the Department of Health in South Africa.^6,7^ The tool extracted process and outcome indicators from the clinical files.

A pilot study of 30 participants enabled problems with the data collection to be identified, and the study’s instruments were modified to meet the study aim and objectives. Participants with glycated haemoglobin (HbA1c) level of 7% or greater were considered to be poorly controlled (efficacy indicator). Signed written informed consent was obtained from each study participant, including access to their medical records.

Data were captured on an MS Excel spreadsheet and analysed using Statistical Package for the Social Sciences (SPSS) version 24 with the assistance of a biostatistician from the University of KwaZulu-Natal. Data are presented as means with standard deviations (s.d.), medians with interquartile ranges (IQRs) for continuous variables, and proportions (%) for discrete variables. Pearson’s chi-square tests and independent samples t-test were used to examine the associations and differences between subgroups. Variables included demographic data (gender, age, educational level, employment status), clinical data (height, weight, body mass index [BMI], comorbidities, blood investigations) and knowledge, attitudes and practices of participants. Association and differences were considered statistically significant at a *p*-value < 0.05. A binomial test was run to determine if the more significant proportion of the participants had measured outcome indicators and a Cronbach’s alpha was run to examine the instrument’s reliability in measuring the participants’ perception of care.

### Ethical considerations

Approval for the study was obtained from the Biomedical Research Ethics Committee of the University of KwaZulu-Natal (reference BE448/19), the provincial Department of Health and the hospital chief executive officer.

## Results

The demographic and lifestyle profile of the participants are presented in Table 1, which shows that most (58.6%) participants were aged 51 to 70 years, with an average age (s.d.) of 59.3 (13.0) years. They were predominantly females (65.3%), African people (30.0%) and Indian people (38.6%), while two-thirds (69.4%) had secondary school education. One-fifth (20%) smoked an average (s.d.) of 14.8 (11.2) pack per years and 22% were current alcohol users, consuming an average of 4.0 units/week, with a maximum consumption of 30 units/week.

**TABLE 1:** Demographic and lifestyle profile of participants (*N* = 360).

Variables	Categories	*n*	%	Mean ± s.d.	Min	Max
Age (years)	≤ 30	12	3.3	-	-	-
	31–40	14	3.9	-	-	-
	41–50	52	14.4	-	-	-
	51–60	113	31.4	-	-	-
	61–70	98	27.2	-	-	-
	Above 70	71	19.7	-	-	-
	Years	-	-	59.3 ± 13.0	18	89
Gender	Male	125	34.7	-	-	-
	Female	235	65.3	-	-	-
Race	African people	108	30.0	-	-	-
	Indian people	139	38.6	-	-	-
	Mixed race people	84	23.3	-	-	-
	White people	25	6.9	-	-	-
	Others	4	1.1	-	-	-
Education	Nil	3	0.8	-	-	-
	Primary	75	20.8	-	-	-
	Secondary	250	69.4	-	-	-
	Tertiary	25	6.9	-	-	-
	Unspecified	7	1.9	-	-	-
Current smoker	Yes	72	20.0	-	-	-
	No	288	80.0	-	-	-
	Pack-years		-	14.81 ± 11.2	1	48
Ex-smoker	Yes	48	13.3	-	-	-
	No	312	86.7	-	-	-
	Pack-years	-	-	20.98 ± 18.5	0.5	70
Alcohol consumption	Yes	77	21.3	-	-	-
	No	284	78.7	-	-	-
	Units consumed/week	-	-	4.0 ± 4.2	1	30
Participation in recreational drug use	Yes	7	1.9	-	-	-
	No	353	98.1	-	-	-

s.d., standard deviation; Min, minimum; Max, maximum.

**FIGURE 1:**
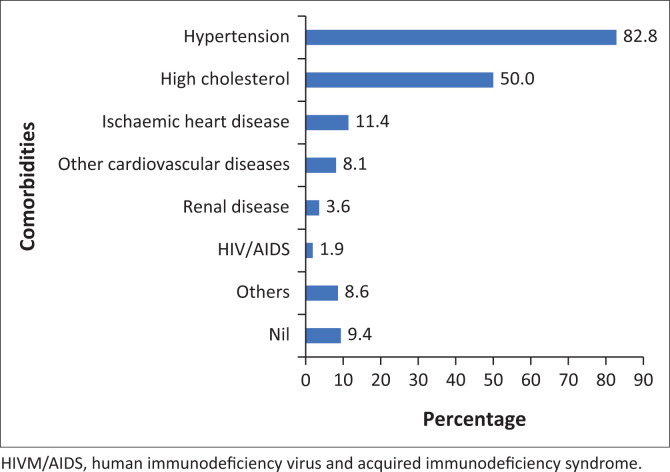
Comorbidities among participants with diabetes mellitus.

Figure 1 presents the associated comorbidities among the participants. The predominant comorbidities among the participants were hypertension (82.8%) and hypercholesterolemia (50.0%).

Renal impairment includes all patients with estimated glomerular filtration rates of less than 60 mL/kg per 1.73 m^2^.

Participants’ knowledge of their DM is presented in Table 2. Most (76.4%) participants reported that they mainly received education on DM from doctors. Most (60.3%) respondents were diagnosed with DM in the last 10 years, and 85.3% of participants have been receiving treatment for DM for the past 10 years, with the mean (s.d.) duration of therapy being 6.4 (5.5) years. Approximately half (51.9%) of the participants did not exercise, while 28.3% did so twice a week. Approximately 89% of respondents do not know their latest HbA1c values. Most (48.1%) of the respondents were on oral treatment only, while 20.8% were on oral treatment and injectables. Ninety percent of the participants had no DM diary, though a similar proportion (94.2%) confirmed that their healthcare professional had spoken to them about their diet. Many reported hypoglycaemia (36.4%). Other medication side effects included gastric-related (16.7%), loss of energy (5.8%) and headache (9.2%).

**TABLE 2:** Participants’ knowledge of their diabetes mellitus (*N* = 360).

Variables	Categories	*n*	%	Mean ± s.d.	Min	Max	Median	IQR
DM education source	Doctor	275	76.4	-	-	-	-	-
	Nurse	41	11.4	-	-	-	-	-
	Dietician	34	9.4	-	-	-	-	-
	Others	10	2.8	-	-	-	-	-
	0–5	100	27.8	-	-	-	-	-
	6–10	117	32.5	-	-	-	-	-
	11–15	61	16.9	-	-	-	-	-
Years since first diagnosis	16–20	21	5.8	-	-	-	-	-
	21–25	36	10.0	-	-	-	-	-
	Above 25	25	7.0	-	-	-	-	-
	Years	-	-	10.9 ± 8.1	1	43	-	-
Number of years treated at the hospital for DM	0–5	224	62.2	-	-	-	-	-
	6–10	83	23.1	-	-	-	-	-
	11–15	29	8.1	-	-	-	-	-
	Above 15	24	6.7	-	-	-	-	-
	Years	-	-	-	1	37	5	2–9
Exercise frequency	Not at all	187	51.9	-	-	-	-	-
	Twice a week	102	28.3	-	-	-	-	-
	3–4 times per week	43	11.9	-	-	-	-	-
	≥ 5 times per week	28	7.8	-	-	-	-	-
	Diet only	5	1.4	-	-	-	-	-
	Tablets only	173	48.1	-	-	-	-	-
Current medical management of DM	Injectables only	107	29.1	-	-	-	-	-
	Tablets and injectables	75	20.8	-	-	-	-	-
History of episodes of low blood sugar (< 4 mmol)	Yes	131	36.4	-	-	-	-	-
	No	229	63.6	-	-	-	-	-
History of episodes of high blood sugar (> 10 mmol)	Yes	223	61.9	-	-	-	-	-
	No	137	38.1	-	-	-	-	-
I know my current/latest HbA1c value	≤ 6.5	5	1.4	-	-	-	-	-
	6.5–7.5	10	2.8	-	-	-	-	-
	7.5–9.5	13	3.6	-	-	-	-	-
	> 9.5	13	3.6	-	-	-	-	-
	Don’t know	319	88.6	-	-	-	-	-
History of cholesterol checks in the past year	No	97	30.0	-	-	-	-	-
	Yes, and low	29	8.1	-	-	-	-	-
	Yes, and high	104	28.9	-	-	-	-	-
	Yes, and normal	130	36.1	-	-	-	-	-
History of the previous ECG done	Yearly	32	8.9	-	-	-	-	-
	Maybe once	184	51.1	-	-	-	-	-
	Never	144	40.0	-	-	-	-	-
History of previous urine test	At every visit	21	5.8	-	-	-	-	-
	Yes, sometimes	156	43.3	-	-	-	-	-
	Yes, but only when sugar is high	120	33.3	-	-	-	-	-
	Never	63	17.5	-	-	-	-	-
I have a DM diary	Yes	36	10.0	-	-	-	-	-
	No	324	90.0	-	-	-	-	-
Has your healthcare worker spoken to you about your diet?	Yes	339	94.2	-	-	-	-	-
	No	21	5.8	-	-	-	-	-

DM, diabetes mellitus; ECG, electrocardiogram; HbA1c, glycated haemoglobin; s.d., standard deviation; IQR, interquartile range; Min, minimum; Max, maximum.

Table 3 outlines participants’ perceptions of care from their healthcare provider. The participants’ overall perception was significantly (*p* < 0.05) associated with their primary DM educator and care provider. The participants reported that they received education on DM and care from doctors, significantly influencing their perception of the care received. Generally, participants were pleased with the care received.

**TABLE 3:** Association between the patient perception of quality of care and diabetes mellitus educator/care provider.

Perception	Major DM educator and care provider										Chi-square test	
	Doctor		Nurse		Dietician		Others		Total		*χ* ^2^	*p*
	*n*	%	*n*	%	*n*	%	*n*	%	*n*	%		
**The doctor/HCW adequately counselled me on my condition**	-	-	-	-	-	-	-	-	-	-	92.022	≤ 0.001*
Strongly disagree	1	20.0	1	20.0	1	20.0	2	40.0	5	1.4	-	-
Disagree	22	75.9	1	3.4	4	13.8	2	6.9	29	8	-	-
Neutral	21	55.3	5	13.2	6	15.8	6	15.8	38	10.5	-	-
Agree	216	82.4	32	12.2	14	5.3	-	-	262	72.6	-	-
Strongly agree	15	57.7	2	7.7	9	34.6	-	-	26	7.2	-	-
**The doctor/HCW involved me in my management goals and treatment options**	-	-	-	-	-	-	-	-	-	-	41.882	≤ 0.001*
Strongly disagree	8	72.7	-	-	2	18.2	1	9.1	11	30.6	-	-
Disagree	62	67.4	9	9.8	15	16.3	6	6.5	92	25.5	-	-
Neutral	18	81.8	3	13.6	-	-	1	4.5	22	6.1	-	-
Agree	181	80.8	29	12.9	12	5.4	2	0.9	224	62.0	-	-
Strongly agree	6	54.5	-	-	5	45.5	-	-	11	3.0	-	-
**The doctor/HCW addressed my concerns**	-	-	-	-	-	-	-	-	-	-	29.610	≤ 0.003*
Strongly disagree	6	71.4	-	-	1	14.3	1	14.3	7	1.9	-	-
Disagree	31	72.1	5	11.6	5	11.6	2	4.7	43	11.9	-	-
Neutral	27	65.9	4	9.8	6	14.6	4	9.8	41	11.4	-	-
Agree	196	80.7	29	11.9	15	6.2	3	1.2	243	67.3	-	-
Strongly agree	16	61.5	3	11.5	7	26.9	-	-	26	7.2	-	-
**After contact with the doctor/HCW, I feel that I understand my condition very well**	-	-	-	-	-	-	-	-	-	-	33.354	≤ 0.001*
Strongly disagree	2	50.0	-	-	2	50.0	-	-	4	1.1	-	-
Disagree	20	83.3	-	-	1	4.2	3	12.5	24	6.6	-	-
Neutral	37	75.5	6	12.2	4	8.2	2	4.1	49	13.6	-	-
Agree	194	76.4	35	13.8	20	7.9	5	2.0	254	70.4	-	-
Strongly agree	22	75.9	-	-	7	24.1	-	-	29	8.0	-	-
**I receive good quality care at WWH**	-	-	-	-	-	-	-	-	-	-	24.665	≤ 0.016*
Strongly disagree	6	66.7	-	-	2	22.2	1	11.1	9	2.5	-	-
Disagree	15	71.4	1	4.8	3	14.3	2	9.5	21	5.8	-	-
Neutral	28	73.7	3	7.9	3	7.9	4	10.5	38	10.5	-	-
Agree	198	78.3	32	12.6	20	7.9	3	1.2	253	70.1	-	-
Strongly agree	28	71.8	5	12.8	6	15.4	-	-	39	10.8	-	-

DM, diabetes mellitus; HCW, healthcare worker; WWH, Wentworth Hospital.

* , Significance at 95% level following a chi-square test.

**FIGURE 2:**
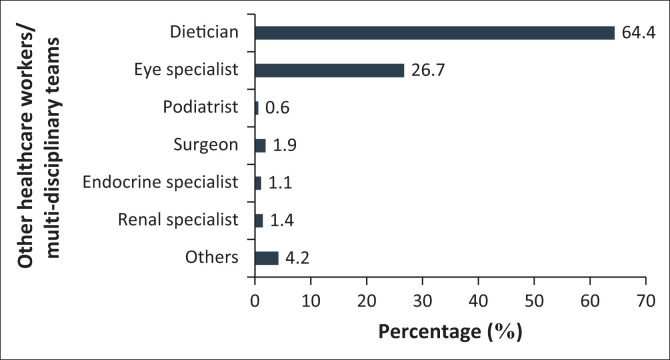
Referral to other healthcare workers in the previous 12 months.

A Cronbach’s alpha to examine the instrument’s reliability in measuring the participants’ perception of care was 0.8, indicating a strong internal consistency of the tool. Figure 2 presents the referral of participants to other healthcare providers in the past 12 months. In the previous 12 months, 64.4% of the respondents were referred to dieticians, 26.7% to eye specialists and 0.6% visited a podiatrist.

**FIGURE 3:**
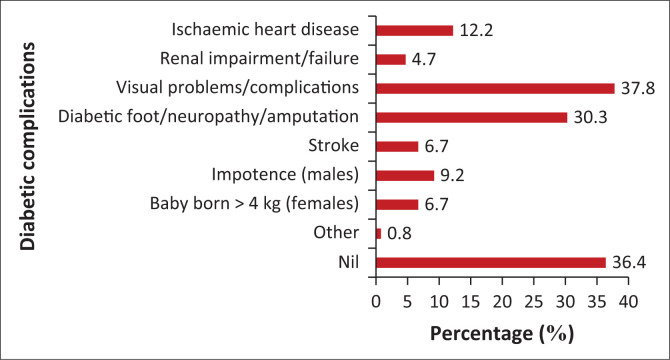
Complications among the participants.

Figure 3 depicts the complications associated with living with T2DM, and 63.6% of participants reported complications, the commonest being visual problems (37.8%) and DM foot disease (30.3%).

Table 4 presents a summary of outcome indicators. Data were extracted from the patient’s clinical files for this analysis. A binomial test was run to determine if the more significant proportion of the participants had measured outcome indicators. A high (72.0%) proportion of the participants had a significant likelihood (*p* < 0.05) of their height not being taken when they visited the hospital (*p* < 0.001). On the other hand, a greater percentage of the participants (95.0%) had a significant tendency for their weight to be measured (*p* < 0.001) during visitation. Similar proportions of the participants had a marked tendency for the BMI (95.3%) and waist circumference (96.9%) not to be documented (*p* < 0.001). Other parameters such as blood pressure, random blood sugar and HbA1c had a significant likelihood of being measured. The mean weight (s.d.) was 81.3 (17.5) kg, the mean blood pressure (s.d.) was 139.5/77.5 (16.1/9.9) millimetres of mercury (mmHg), and the mean HbA1c (s.d.) was 8.6% (2.4). Twelve percent of participants in this study had HbA1c values greater than 10%. While 28.8% achieved a value of 7 or lower, 71.2% had HbA1c value of 7.1+, above the goal target of 7%.

**TABLE 4:** Outcome and process indicators.

Variable	Documented measurement				*p*	Mean	s.d.	Min.	Max.	Last documented							
	Yes		No							Not at all		Three monthly		Six monthly		Annually	
	*n*	%	*n*	%						*n*	%	*n*	%	*n*	%	*n*	%
Weight (kg)	342	95.0	18	5.0	< 0.001*	81.3	17.5	44.0	140.0	21	5.8	61	16.9	247	68.6	31	8.6
BMI (kg/m^2^)	19	4.7	343	95.3	< 0.001*	31.5	10.4	19.0	54.0	343	95.3	3	0.8	14	3.9	-	-
Waist circumference (cm)	11	3.1	349	96.9	< 0.001*	99.6	18.0	73.0	130.0	349	96.9	5	1.4	6	1.7	-	-
Blood pressure (mmHg)	358	99.4	2	0.6	< 0.001*	139.5/77.7	16.1/9.9	100.0/51.0	193.0/111.0	8	2.2	69	19.2	253	70.3	30	8.3
Random blood sugar (mmol/L)	357	99.2	3	0.8	< 0.001*	7.8	2.6	1.00	18.8	8	2.3	69	19.2	253	70.3	30	8.3
HbA1c	321	89.2	39	10.8	< 0.001*	8.6	2.4	5.60	19.1	42	11.7	55	15.3	238	66.1	25	6.9
Foot examinations	9	2.5	351	97.5	< 0.001*	Abnormal	-	-	-	351	97.5	4	1.1	3	0.8	2	0.6
Urine dipstick	36	10.0	324	90.0	< 0.001*	1+ Pr (50%)	-	No Pr	3+	324	90.0	9	2.5	23	6.4	4	1.1

s.d., standard deviation; Pr, protein; HbA1c, glycated haemoglobin; Min., minimum; Max., maximum.

* , Significance at 95% level following a binomial test.

## Discussion

The study found some promising and alarming findings. The overall QOC was suboptimal but patients’ perception of their care at the facility was generally regarded as very good. However, this did not translate into better lifestyle practices, better outcome indicators and fewer complications.

The dominant representation of women among the study participants reflects the overall higher level of usage of health facilities by women in South Africa.^13^ The patients included in this study had an overall high level of education, but this should, in theory, have had an impact on their knowledge of T2DM glycaemic control. This study showed that educational level might not be a good predictor of better therapeutic compliance, similar to previous studies.^14^ Sources of DM education are crucial for gaining relevant lifestyle changes required to improve glycaemic control. Patient education and motivation are crucial to improve compliance with medications. Education provided by a trained diabetes educator who focuses on improving behaviour is more significant for good glycaemic control.^15^ Inadequate staffing with doctors and nurses and a lack of DM educators in this facility could be reasons for the poor lifestyle practices.^16^ Twenty per cent of the respondents were current smokers at the time of the study. The predominant comorbidity conditions among the respondents were hypertension and hypercholesterolemia. This finding is similar to studies conducted among the outpatients’ population and a cross-section of adults, which reported comorbidity of T2DM and hypertension.^17,18^ Most participants were on oral treatment only for the medical management of their T2DM, comparable to a local study within the same district.^19^ More than half of the participants indicated that they do not exercise frequently. These findings are concerning because there is strong evidence that lifestyle modifications such as physical activity and smoking cessation provide benefits in controlling T2DM and preventing complications.^20,21^

A high proportion of our participants felt they understood their condition very well after contacting the healthcare professionals. Similarly, a high percentage of participants thought that the QOC they received was exemplary and that they were satisfied with the healthcare. This finding is similar to a study conducted in the Cape Metropolitan district of the Western Cape, South Africa, which reported a high perceived level and satisfaction with the QOC.^22^ These findings revealed much higher satisfaction scores than studies conducted in the United Kingdom, India, Kosovo, Iraq and Botswana, where the satisfaction rate ranged from 50% to 70%.^22^ Patient experience of care must be linked to outcome indicators to indicate a better QOC. Patients’ perception of care and satisfaction are part of the quality assurance process and have become globally integral to measuring healthcare quality. Patients’ perception of care responses may have been influenced by the principal investigator who is a doctor working at the institution.

Most guidelines recommend a multidisciplinary approach to chronic disease management, and this was lacking in this study. The dietician and ophthalmologists were suboptimally utilised despite a very large number of uncontrolled participants with T2DM. A large number of patients with visual and foot problems further emphasise the need for earlier and continued MDT involvement.^7^

Efficacy indicators, which included outcome and process indicators, showed alarming figures. This study’s prevalence of uncontrolled T2DM is higher than similar findings from studies conducted in the Western Cape province and North West province of South Africa,^23^ but comparable with another South African report from KwaZulu-Natal province by Igbojiaku and colleagues.^24^ The high prevalence rate of uncontrolled T2DM observed in our sample is worrisome, given the harmful health implications of uncontrolled T2DM. Several underlying issues may contribute to uncontrolled T2DM among the population studied. It might be possible that many participants do not truly understand the health implications of having uncontrolled T2DM.^7^ Further clarity on the suboptimal glycaemic control in participants on insulin therapy could not be ascertained due to a lack of data on the timing of initiation and dosing schedule of insulin therapy in the participants. Nevertheless, the possibility of clinical inertia, such as delays in initiating insulin therapy, failure to optimise insulin doses and inadequate follow-up of participants, cannot be ignored as the reasons for our results.^19^ In addition to clinical inertia, access to clinic follow-up for optimisation of insulin doses may probably be unattainable due to the costs of transport to the hospital due to poverty. Newer effective oral agents are not available at public sector facilities in KwaZulu-Natal.^7^ Participants did not have routine access to an entire multidisciplinary team of healthcare professionals (e.g. dietitians, podiatrists) and were restricted to EDL medications, which are often liable to substitution by the dispensary.^6,25^

Our findings of suboptimal care for T2DM are consistent with previous studies conducted in America and South Africa.^23,26,27,28^ Glycated haemoglobin levels were documented in 89.2%, a significant proportion of participants, despite 81.4% of patients attaining a minimum standard measurement as compared to the 56% of patients in the American study of QOC provided to DM patients.^27^ Twelve per cent of participants in this study had HbA1c greater than 10%. In comparison 71.2% had a value of 7.1+ (goal target of 7%) in contrast to a previous South African study done in 2016 of 83.8% in the OR Tambo District.^23^ The mean HbA1c of newly diagnosed patients in good general health has been adjusted to a new target of less than 6.5%.^7^ This may be seen as an elusive target if 88.6% of patients do not know their HbA1c levels despite the national guidelines highlighting a need to involve the patient in the discussion about setting glycaemic goals.^7^ Glycaemic targets are based on the duration of their T2DM, general health status, life expectancy and risk of hypoglycaemia,^7^ which contrasts with the participant’s perception of the QOC received; 78.6% of participants felt that they knew their condition well and 81% felt they received good QOC.

The physical and other health outcome examinations in the facility need to be reviewed. Foot examinations (which should be performed at least annually, more frequently in those with high risk for ulcers) were not documented in 97.5% of participants. Similarly, findings of nondocumentation of 94% were found in the United States.^27^ This is concerning because, after examination, the patient’s risk stratification should be determined and recorded, highlighting the individual’s need for foot examinations to be performed at each regular visit and alludes to a globally poorly achieved indicator and requires review by all worldwide. Urine protein measurements were not performed in 90% of participants compared to the American study of 52%.^18^ A total of 4.7% of participants had renal impairment as a complication, of which only 1.4% had been referred to a renal specialist in the past 12 months. Some participants had already developed complications such as stroke, ischaemic heart disease and renal failure. This is, however, not surprising based on the high prevalence of uncontrolled T2DM. Additionally, an analysis of annual cholesterol screening demonstrated a rate of 63% compared to our study’s findings of 75%.^26^ Annually, one out of four participants was not monitored for lipid abnormalities, and of those tested, 50% had elevated cholesterol levels.

Many participants’ BMIs were not recorded due to poorly recorded heights. Obesity is an independent determinant of uncontrolled T2DM.^29^ This is a major gap, as many other studies have reported an association between obesity and uncontrolled T2DM.^30^ The physical inactivity of most participants did not achieve the recommended physical activity guidelines. Physical inactivity was an independent and significant determinant of uncontrolled T2DM in the study. The benefits of exercise in reducing cardiovascular risks have been well-documented.^31,32,33,34^ Given the tremendous toll lifestyle factors have on the health of participants with T2DM, ongoing efforts are needed to address and change the societal determinants at the root of these problems.

Despite the frequency of primary care provider visits for many participants during the year, T2DM management was inadequate. This lack of adequate preventive care could lead to an increased risk of developing acute and chronic complications of T2DM, creating an even more significant future burden on the healthcare system and negative consequences for patients. While external factors such as lack of time and patient noncompliance are perceived as essential issues, it is important to note that physician-related factors continue to be an issue, including lack of familiarity with guidelines and implementation of guideline recommendations.^26,27^ We did not assess physician-reported barriers to guideline adherence for specific aspects of care. It is well-documented that preventative and holistic QOC, and achieving reasonable glycaemic control, minimises the micro- and macrovascular complications of T2DM, leading to a better quality of life,^35,36,37^ and decreases the negative consequences for both the patients and the healthcare system.^38^ There is, therefore, a great need to improve the QOC for patients with T2DM and other NCDs, as most South African patients access health services at primary healthcare clinics and district hospitals, which are overstretched and underresourced, thereby putting a strain on the facilities.^39^ A study into glycaemic control in an urban public sector’s primary level care in Cape Town, South Africa, found that only 49.4% of participants achieved their glycaemic target,^40^ which contrasted dramatically with the 15.7% who achieved a target of having HbA1c of less than 7% in a rural district hospital in Hlabisa, northern KwaZulu-Natal province.^41^ This contrast between the urban and rural areas is of concern and may indicate healthcare system challenges and/or failures.

The Department of Health has provided a national core standard quality assessment framework for facilities to use; this audit is used to collect baseline data for quality improvement, patient-centered care and compliance to quality standards. Most facilities use this framework to assess different areas of the hospital’s services, for example, the outpatient department in its entirety but does not include disease-specific auditing and reporting.^42,43^ It is up to each facility to continuously assess and initiate quality improvement plans from gaps identified and expand the framework in each department as needed. Priority attention is needed to ensure that all facilities provide a comprehensive range of services and QOC, especially in light of the PHC re-engineering, grouping and amalgamation of data using systematic review methods are effective strategies to inform health planning and policy making.^42,43,44^

Substantial quality gaps still exist and persist in the management of T2DM. The impact of this rapidly emerging health burden can be minimised through effective management of the supply-sensitive services (frequency of visits, tests, imaging, time in hospital, and aggressive use of services at the end of life), continuity of care and use of an integrated chronic care model.^23,27,31^ Patient-centred communication that incorporates patient preferences, assesses literacy and numeracy, and addresses cultural barriers to care should be used.^35,45^ Care should be aligned with components of the chronic care model to ensure productive interactions between a prepared, proactive team and an informed, activated patient.^46^ When patients are not meeting treatment goals, ‘reassessing the treatment regimen may require evaluating barriers such as income, health literacy, DM-related distress, depression, poverty and competing demands, including those related to family responsibilities and dynamics’.^47^ There is an urgent need to re-engineer primary health care by prioritising T2DM care and other NCDs.

### Recommendations

Performance feedback, physician reminders and structured care management plans are linked with better care processes. Future work should focus on improving the design of clinical decision support tools and combining these tools with other methods for enhancing the quality, such as electronic reminders and medical record systems with integrated laboratory and medication data. A monitoring plan backed by necessary funding to raise public awareness, risk reduction and availability of essential medication should be provided in all community sectors. An all-encompassing approach is critical for a better QOC.

## Conclusion

This study indicates that the QOC was suboptimal due to poor efficacy indicators, poor knowledge and lack of adequate lifestyle measures, despite the frequency of medical practitioner reviews. With the realities of resource constraints in South Africa’s public sector, health professionals should strive to attain the key domains of QOC as outlined in the guidelines. Management should be safe, effective, patient-centred, timely, efficient and equitable. Achieving a lasting quality improvement system in healthcare seems to be a demanding challenge.
